# Melatonin contributes to the hypertrophic differentiation of mesenchymal stem cell-derived chondrocytes via activation of the Wnt/β-catenin signaling pathway: Melatonin promotes MSC-derived chondrocytes hypertrophy

**DOI:** 10.1186/s13287-021-02536-x

**Published:** 2021-08-21

**Authors:** Xuan Wang, Tianwei He, Lei He, Bu Yang, Zhongyu Liu, Mao Pang, Peigen Xie, Liangming Zhang, Limin Rong

**Affiliations:** 1 Department of Spine Surgery, The Third Affiliated Hospital of Sun Yat-Sen University, Guangzhou, People’s Republic of China; 2 Guangdong Provincial Center for Quality Control of Minimally Invasive Spine Surgery, Guangzhou, People’s Republic of China; 3 Guangdong Provincial Center for Engineering and Technology Research of Minimally Invasive Spine Surgery, Guangzhou, People’s Republic of China

**Keywords:** Melatonin, Mesenchymal stem cells, Chondrocyte hypertrophy, Wnt/β-catenin

## Abstract

**Background:**

Hypertrophy is a critical process for chondrocyte differentiation and maturation during endochondral ossification, which is responsible for the formation of long bone and postnatal longitudinal growth. Increasing evidence suggests that melatonin, an indole hormone, plays a pivotal role in chondrogenesis. However, little is known about the effects of melatonin on the terminal differentiation of chondrocytes.

**Methods:**

Mesenchymal stem cell (MSC)-derived chondrocytes generated by a high-density micromass culture system were induced to undergo hypertrophic differentiation. Melatonin-mediated hypertrophic differentiation was examined by reverse transcription polymerase chain reaction analysis (RT-PCR) analysis, histological staining and immunohistochemistry. Activation of the Wnt signaling pathway was evaluated by PCR array, RT-PCR, western blotting and immunofluorescence. XAV-939, a Wnt signaling pathway antagonist, was further used to determine whether the effect of melatonin on chondrocyte hypertrophic differentiation was mediated occurred by activation of Wnt signaling pathway.

**Results:**

Histological staining showed melatonin increased chondrocyte cell volume and the expression of type X collagen but decreased the expression of type II collagen compared with the control group. RT-PCR showed that melatonin significantly up-regulated the gene expressions of biomarkers of hypertrophic chondrocytes, including type X collagen, alkaline phosphatase, runt-related transcription factor 2, Indian hedgehog and parathyroid hormone-related protein receptor, and melatonin down-regulated the mRNA expression of hallmarks of chondrocytes, including parathyroid hormone-related protein. PCR array showed that the effect of melatonin on chondrocyte hypertrophic differentiation was accompanied by the up-regulation of multiple target genes of the canonical Wnt signaling pathway, and this effect was blocked by XAV-939.

**Conclusions:**

The current findings demonstrate that melatonin enhances the hypertrophic differentiation of MSC-derived chondrocytes through the Wnt signaling pathway. Our findings add evidence to the role of melatonin in promoting bone development and highlight the positive effects of melatonin on terminal differentiation of chondrocytes.

**Supplementary Information:**

The online version contains supplementary material available at 10.1186/s13287-021-02536-x.

## Introduction

Endochondral ossification (EO), one of the main processes in skeletal development in vertebrates, involves the progressive replacement of cartilaginous tissues by bony tissues for normal bone architecture formation [1, 2]. Mesenchymal stem cells (MSCs), the progenitors of chondrocytes, undergo a series of steps including proliferation, pre-hypertrophy and hypertrophy during EO for cell maturation and skeletal morphogenesis. Longitudinal bone growth occurs at the growth plate, an organized cartilaginous structure in the metaphysis [3]. The growth plate is subdivided into three structurally and functionally independent zones: the resting zone, which contains round chondrocytes; the proliferative zone, which contains flattened chondrocytes; and the hypertrophic zone, which contains enlarged chondrocytes. These zones are spatially located from both ends to the middle along the vertical axis of the long bone [4, 5]. The hypertrophic zone serves as a signal-transducing center, in which several cell signaling pathways cross-talk with each other for the precise regulation of chondrocyte hypertrophic differentiation [5]. Chondrocyte hypertrophy contributes more substantially to bone growth rate than chondrocyte proliferation through promoting enlargement of cell volume [6, 7]. Moreover, hypertrophic chondrocytes act as “intermediators” in the turnover of cartilage to bone. Therefore, chondrocyte hypertrophic differentiation has been highlighted as the pivotal stage in EO.

Hypertrophy, an irreversible stage of chondrocyte terminal differentiation, is controlled by several signaling pathways, including the Wnt/β-catenin signaling pathway [8–10]. The canonical Wnt/β-catenin pathway orchestrates chondrocyte hypertrophy by the translocation of β-catenin into the nucleus and β-catenin binding with T cell-specific factor (TCF) or lymphoid enhancer binding protein (LEF) [11], leading to activation of the hypertrophy-related RUNX2 gene in the nucleus [12]. However, the concrete molecular framework for chondrocyte hypertrophy remains unclear.

Melatonin, an amine hormone, is mainly secreted by the pineal gland in mammals, especially in dark environments. Melatonin elicits a variety of biological functions via membrane and nuclear receptors and plays vital roles in the regulation of many pathophysiological processes [13] such as clock rhythm [14, 15], immune response [16, 17], cellular senescence [18, 19], and bone development [20, 21]. Several studies have shown that melatonin also participates in chondrocyte differentiation and maintenance. Our previous data showed that melatonin at a lower concentration (nanomolar level) promotes the differentiation of MSCs into chondrocytes [22]. Pei et al. reported that treatment of porcine joint chondrocytes with a lower concentration’s melatonin enhances the synthesis of matrix, while a higher concentration’s melatonin resulted in down-regulation of cartilage gene expression [23]. Zhong ZM et al. also showed that melatonin at a high concentration (micromolar level) inhibits the proliferation and differentiation of chondrocytes in the growth plate of the vertebral body in rats [24]. These results indicate that high concentrations of melatonin may have deleterious effects on the differentiation of chondrocytes; however, melatonin at a moderate concentration may be beneficial to chondrocyte differentiation and maintenance. Although studies have shown that melatonin participates in chondrocyte differentiation, its effect on hypertrophic differentiation of chondrocytes is unknown.

In the present study, we evaluated the effect of melatonin at a moderate concentration (100 nM) on human bone marrow mesenchymal stem cells (BMSCs) and C3H10T1/2-derived chondrocytes and further analyzed the effect of melatonin on EO. Our study aim is to provide insights into understanding skeletal development and the potential use of melatonin as a therapeutic drug for cartilage disorders and cartilage development-related diseases.

## Materials and methods

### Cell culture and reagents

This study was approved by the ethics committee of Sun Yat-Sen University, and informed consent was obtained from all the individuals included in this study. Bone marrow samples were obtained from five human volunteers (mean age: 25 years, range: 18–30 years). MSCs were cultured by the whole bone marrow culture method [25], which fulfills the criteria proposed by the International Society for Cellular Therapy (ISCT) [26] (see Additional file 1). We used the murine mesenchymal stem cell line C3H10T1/2 (Cell Bank of the Chinese Academy of Science, Shanghai, China) in some experiments to eliminate the influence of MSC heterogeneity and acquire more solid information. The cell lines were cultured in 25 cm^2^ flasks containing 5 ml low-glucose Dulbecco’s modified Eagle’s medium (DMEM) with 10% fetal bovine serum (FBS) at 37 °C with 5% CO_2_. The growth medium was changed every three days. For cell passage, the cells were digested with trypsin at 80% confluence and then subcultured in flasks. After monolayer expansion, cells from passage 3–6 were selected for experiments.

In some of these experiments, the β-catenin antagonist XAV-939 (10 μM) (Selleckchem, USA) was used to block the Wnt/β-catenin signaling pathway and the agonist CHIR-90021 (5 μM) (Selleckchem, USA).

### Chondrogenic and hypertrophic differentiation

A high-density micromass culture system was used for the chondrogenic differentiation of MSCs and C3H10T1/2 cells, as previously described [22, 25]. The cells were trypsinized, washed with PBS, and resuspended at 2 × 10^7^ cells/mL in chemically defined chondrogenic medium consisting of high-glucose DMEM supplemented with 10 ng/mL recombinant human transforming growth factor–β3 (TGF-β3; Peprotech, USA), 100 nM dexamethasone (Sigma, USA), 50 μg/mL ascorbic acid 2-phosphate (Sigma, USA), 1 mM sodium pyruvate (Sigma, USA), 40 μg/mL proline (Sigma, USA), and ITS + Universal Culture Supplement Premix (Corning, USA; final concentrations: 6.25 μg/mL bovine insulin, 6.25 μg/mL transferrin, 6.25 μg/mL selenous acid, 5.33 μg/mL linoleic acid, and 1.25 mg/mL bovine serum albumin). The cell suspension (15 μL) was carefully placed in each well of a 24-well plate. Cells were cultured in a 37 °C incubator for 2 h, and then, 500 μL of chondrogenic medium was slowly added along the wall of the well. The cell aggregates were cultured in chondrogenic medium for two weeks, and the medium was changed every other day. The medium was changed to hypertrophic-enhancing medium by the following steps: withdrawal of TGF-β from the chondrogenic medium, addition of 1 nM triiodothyronine (T3) (Sigma, USA), reduction of dexamethasone to 1 nM, and addition of vehicle or 100 nM melatonin (Sigma, USA). We confirmed that melatonin at 100 nM had no deleterious effects on MSCs (see Additional files 2–4). The induced hypertrophic cartilage tissues were harvested after 1–3 weeks. CHIR99021 a Wnt signaling agonist was used to activiate the Wnt/β-catenin signaling pathway for determining the effect of Wnt on chondrocytes hypertrophy (see Additional files 5, 6).

### Reverse transcription and real-time polymerase chain reaction (RT-PCR) analysis

Total RNA was isolated from the aggregates using the total RNA Kit (Omega Bio-Tec, USA) following the manufacturer’s protocol. Total RNA (500 ng) was converted to cDNA using the reverse transcription kit (TaKaRa, JAPAN). All RT-PCRs were performed on a Roche 480 Real-Time PCR Detection System (Roche Diagnostics) in 20 μl reaction volume containing 10 μl of SYBR Green I Master Mix (Roche Diagnostics), 2 μl of 10 mM sense or antisense primer, and 6 μl of RNAse-free water. The expressions of the following genes were examined: type X collagen (COL10A1), alkaline phosphatase (ALP), runt-related transcription factor 2 (RUNX2), Indian hedgehog (IHH), parathyroid hormone-related protein receptor (PTHrP-R), and parathyroid hormone-related protein (PTHrP) mRNAs. Glyceraldehyde-3-phosphate dehydrogenase (GAPDH) mRNA was used for normalization of gene expression. The PCR reaction conditions were as follows: 1 min at 95 °C, followed by 39 amplification cycles of 15 s at 95 °C, 15 s at 60 °C, and 20 s at 72 °C. After the last cycle, a melt curve was generated. The Ct value of GAPDH mRNA was subtracted from the Ct value of the gene of interest (ΔCt). The average ΔCt value of the triplicate reactions was determined. MSCs cultured in the pellet culture system were used as controls. The relative expression level for each gene was expressed as fold change by the 2^−ΔΔCT^ method.

### PCR array

Amplified cDNA was diluted with nuclease-free water and added to the RT^2^ qPCR SYBR green Master Mix (Roche Diagnostics). Next, 25 μl of the experimental cocktail was added to each well of the Wnt signaling pathway PCR array (SA Biosciences, Frederick MD, USA). PCR amplification was performed using RT-PCR analyses as described above. Data were analyzed using the 2^−ΔΔCT^ method and presented as fold change in the target gene normalized to the mean of endogenous housekeeping genes (GAPDH).

### Western blotting

Cell and tissue lysates were lysed using RIPA lysis buffer (Beyotime, China) supplemented with the Halt™ Protease and phosphatase inhibitor cocktail (Thermo, USA). Samples were separated by electrophoresis on 8% SDS–PAGE gels and transferred onto PVDF membranes (Millipore, USA). The membranes were blocked with 5% non-fat milk, followed by incubation with primary antibodies targeting the following proteins, according to the manufacturer’s instructions: TCF1/TCF7, H3, and Actin (Cell Signaling Technology, China). Membranes were then incubated with the appropriate secondary antibodies (Cell Signaling Technology) according to the manufacturer’s instructions. Protein bands were developed and analyzed using an automatic chemiluminescence system (Tanon, China), and the intensity of the bands (pixels/band) was determined using ImageJ software as arbitrary optical density units.

### Histology and immunohistochemistry

The cartilage aggregates were fixed in 10% formaldehyde solution for 24 h, followed by dehydration in an ethanol gradient and embedding in paraffin. The paraffin blocks were cut by a pathologic microtome section processor into sections approximately 3–5 μm thick, and the sections were carefully placed on glass slides. Toluidine blue staining was used to assess proteoglycan levels. Briefly, the sections were dewaxed with xylene and rehydrated with an alcohol gradient; the sections were then exposed to toluidine blue solution for 15 min and then immersed in acetone for 3 min. Immunohistochemistry was performed to evaluate the expressions of collagen type II (Col-II) and collagen type X (Col-X) using the Histostain-Plus kit (ZSGB-BIO, Beijing, China). After dewaxing and rehydration, tissue sections were treated with pepsin at 37 °C for 10 min, incubated with peroxidase-blocking solution for 10 min, and blocked with 5% bovine serum albumin for 30 min at room temperature. The sections were then incubated with the following primary antibodies overnight at 4 °C: mouse anti-human Col-X monoclonal antibodies (Abcam, USA) diluted at 1:2000 and rabbit anti-human Col-II polyclonal antibodies (Abzoom Biolabs, USA) diluted at 1:500. Detection was conducted with a DAB Horseradish Peroxidase Color Development Kit (ZSGB-BIO, Beijing, China) according to the manufacturer’s protocol. Hematoxylin served as a counterstain for the nucleus. After dehydration in alcohol and clearance in xylene, the sections were mounted and photographed with a microscope (Leica, Germany).

### Immunofluorescence

The cells were washed with PBS twice, fixed in 4% paraformaldehyde at room temperature for 15 min, and washed with PBS twice. The cells were then incubated with primary antibodies against MT1, MT2, or β-actin (Abcam, USA) according to the manufacturer’s instructions. The cells were washed and then incubated with species-matched secondary antibodies (Abcam, USA) labeled with Alexa Fluor 488 (Life Science, USA) diluted at 1:500 in 1% BSA according to the manufacturer’s instructions. The nuclei were labeled with Hoechst 33,342 (Beyotime Biotechnology, Beijing, China).

### Statistical analysis

All experiments were performed at least three times. Statistical analysis was performed using one-way ANOVA followed by *t* test with the SPSS version 20.0 software. *P* < 0 0.05 was considered statistically significant.

## Results

### Melatonin promotes hypertrophic differentiation of MSC-derived chondrocytes

**Fig. 1:**
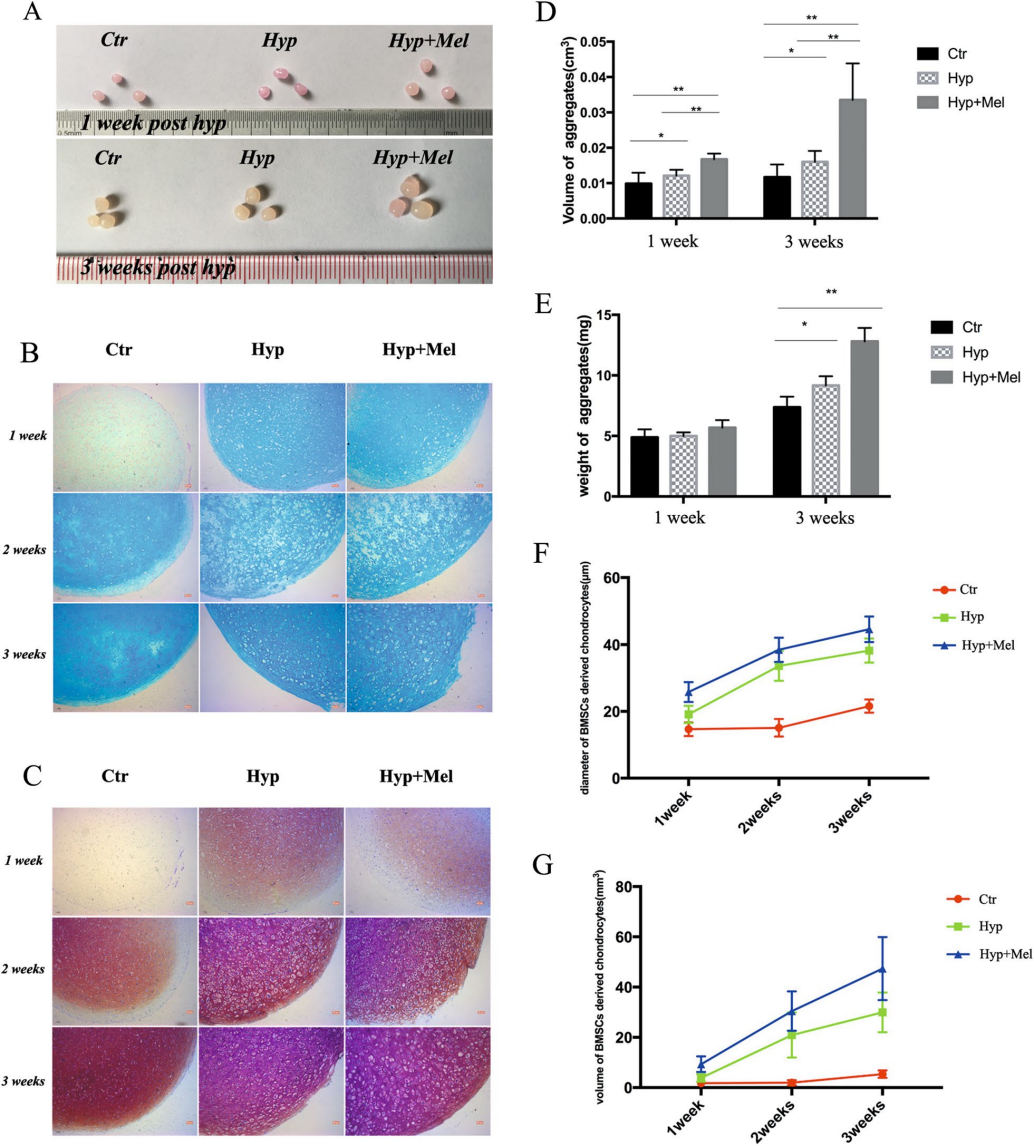
Melatonin promotes hypertrophic differentiation of MSC-derived chondrocytes. **a** Morphologic characteristics of aggregates post-hypertrophic differentiation at week 1 and week 3. **b** Alcian blue staining of chondrocytes during hypertrophic differentiation at week 1, week 2, and week 3. Scale bar = 200 μm. **c** Toluidine blue staining of chondrocytes during hypertrophic differentiation at week 1, week 2, and week 3. Scale bar = 200 μm. **d** Volume of aggregates in the indicated groups at week 1 and week 3. **e** Weight of aggregates in the indicated groups at week 1 and week 3. **f** Diameter of chondrocytes in the indicated groups at week 1, week 2, and week 3. **g** Volume of chondrocytes in the indicated groups at week 1, week 2, and week 3 (volume = 4/3 πR^3^, R = 1/2 diameter). *P < 0.05, **P < 0.01

Hypertrophy contributes to an increase in the volume of an organ or tissue based on the enlargement of component cells. Therefore, to determine the effect of melatonin on hypertrophic chondrocytes, we examined the effect of melatonin on both the volume and weight of hypertrophic BMSC-derived chondrocytes. We induced hypertrophy for 1 week and 3 weeks and found that the melatonin-treated chondrocyte aggregates were much larger in size than those in the control group (Fig. 1a). The aggregates were globoids, and the volumes of aggregates were calculated by measuring the diameter; the dry weight of aggregates was measured with a precision electronic scale. Melatonin remarkably increased both the volume and weight of hypertrophic differentiated BMSC-derived chondrocytes (Fig. 1d, e). Alcian blue and toluidine blue staining were performed to reveal morphological details of single chondrocytes, and chondrocytes in the melatonin treatment group were much larger in size than that without melatonin treatment (Fig. 1b, c). The quantitative results and statistical analyses of chondrocyte diameter and volume are shown in Fig. 1f, g.

### Melatonin promotes hypertrophic gene expression in the chondrogenesis of BMSCs and C3H10T1/2 cells

**Fig. 2:**
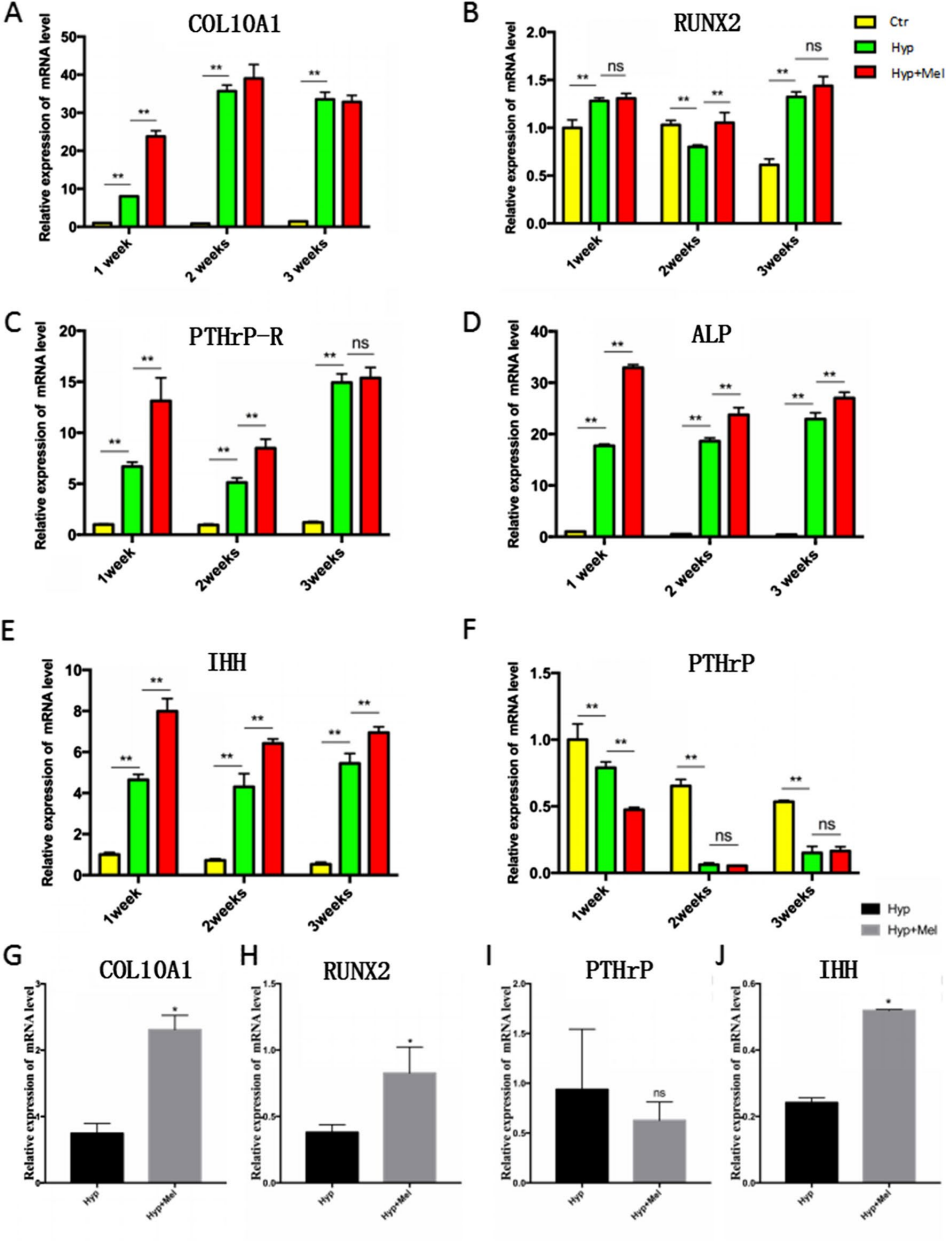
Melatonin promotes hypertrophic gene expression in the stem cells derived chondrocytes. **a**–**f** The mRNA expression of collagen type X (COL10A1) (A), runt-related transcription factor 2 (RUNX2) (**b**), parathyroid hormone-related protein receptor (PTHrP-R) (**c**), alkaline phosphatase (ALP) (**d**), Indian hedgehog (IHH) (**e**), and parathyroid hormone-related protein (PTHrP) (**f**) in BMSC-derived chondrocytes treated as indicated. **g**–**j** The mRNA expression of COL10A1 (**g**), RUNX2 (**h**), PTHrP (**i**), and IHH (**j**) in C3H10T1/2 -derived chondrocytes. Gene expressions were normalized to GAPDH mRNA. *P < 0.05; **P < 0.001; ns, not significant

Chondrocyte hypertrophy is characterized by the up-regulation of COL10A1, IHH, PTHrP-R, RUNX2, and ALP and the down-regulation of PTHrP. We therefore examined the mRNA expression of these factors at different time points during hypertrophic differentiation (Fig. 2a–f). The mRNA levels of all factors, except PTHrP mRNA, were significantly increased in the melatonin treatment group compared with the group without melatonin treatment. We performed a similar assay in C3H10T1/2 cells, and similar results were observed in C3H10T1/2 cell chondrogenesis (Fig. 2g–j).

**Fig. 3:**
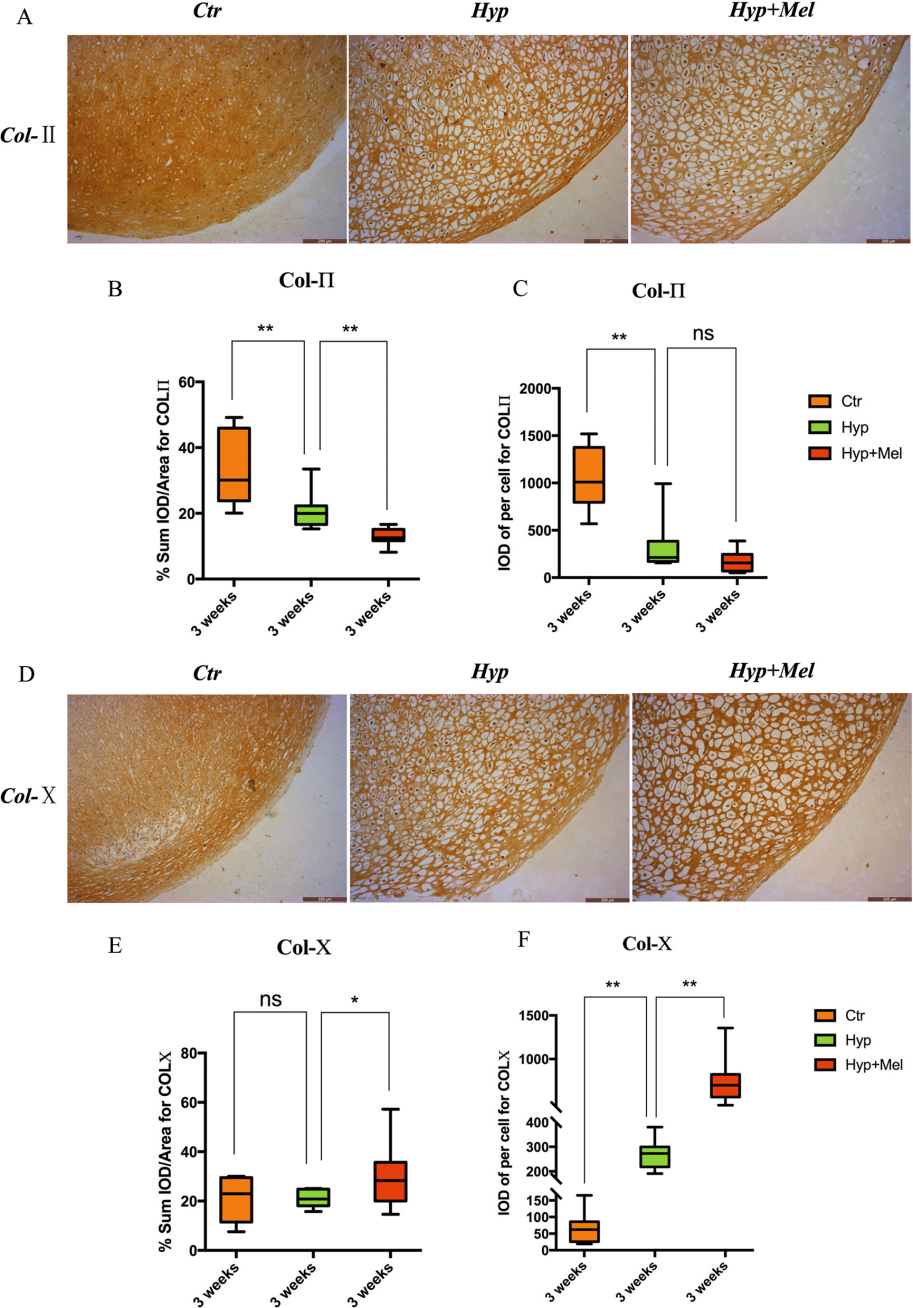
Melatonin decreases collagen-II expression and induces collagen-X expression during hypertrophy. **a** Immunohistochemical staining of Col-II in aggregates after hypertrophy differentiation for 3 weeks. Scale bar = 200 μm. **b** Gray value of Col-II staining in induced cartilage tissues. **c** Gray value for Col-II staining in a single chondrocyte. **d** Immunohistochemical staining of Col-X in aggregates after hypertrophy differentiation for 3 weeks. Scale bar = 200 μm. **e** Gray value of Col-X staining in induced cartilage tissues. **f** Gray value for Col-X staining in a single chondrocyte. *P < 0.05, **P < 0.01

Collagen remodeling, in which Col-II level decreases and Col-X level increases, is a characteristic event during hypertrophic differentiation. Immunohistochemistry of aggregates after hypertrophy differentiation showed that Col-II expression level decreased and Col-X expression level increased in the presence of melatonin (Fig. 3a, d). Integral optical density analysis was performed and demonstrated the positive effect of melatonin on BMSC-derived chondrocyte hypertrophic biomarker expression (Fig. 3b, c, e, f).

### Melatonin membrane receptor inhibitor blocks melatonin-mediated hypertrophic differentiation

**Fig. 4:**
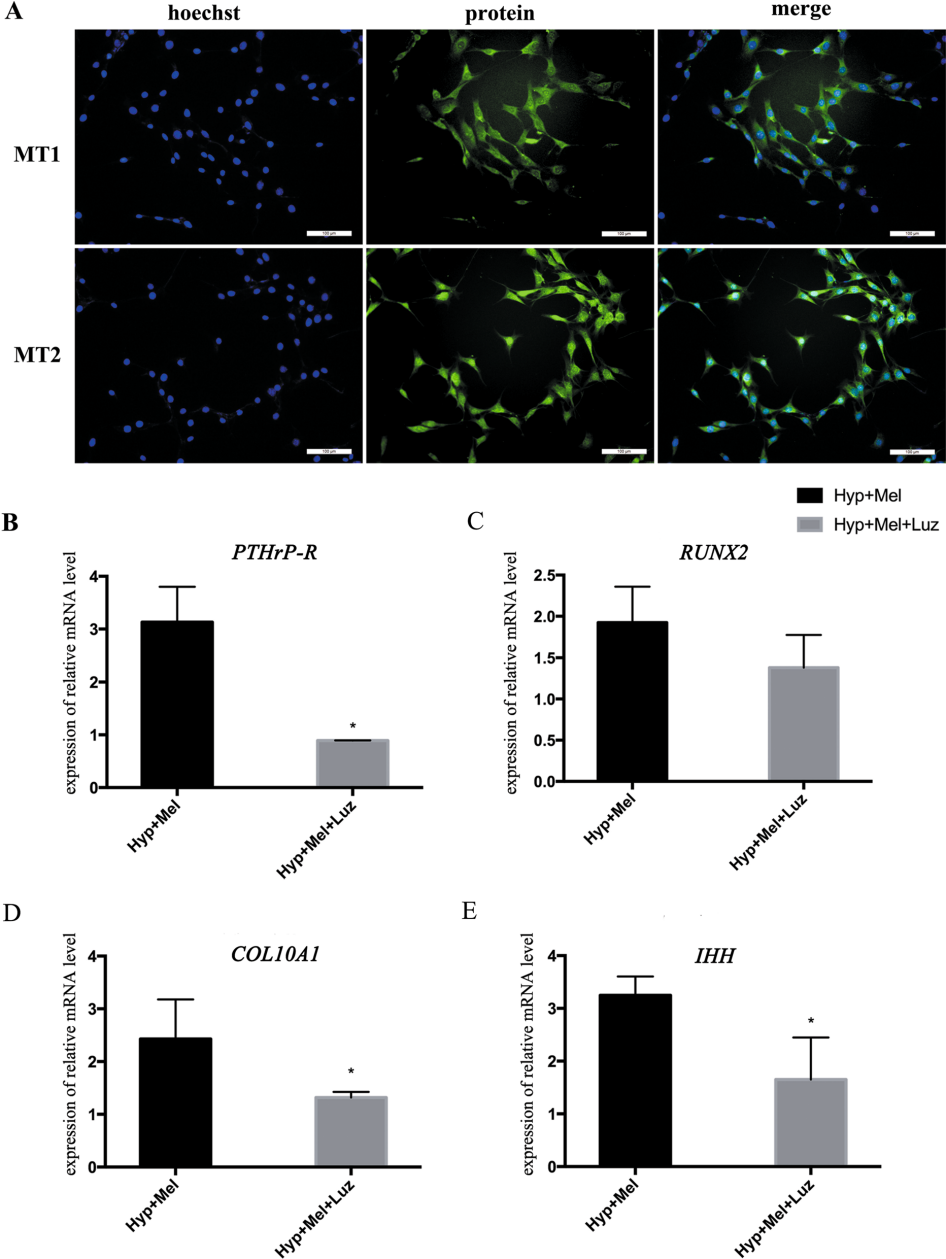
Melatonin membrane receptor inhibitor blocks melatonin-mediated hypertrophy. **a** Immunofluorescence staining of melatonin membrane receptors MT1 and MT2 in C3H10T1/2 cells. **b**–**e** The mRNA expressions of PTHrP-R (**b**), RUNX2 (**c**), COL10A1 (**d**), and IHH (**e**) in C3H10T1/2-derived chondrocytes treated as indicated. Gene expressions were normalized to GAPDH mRNA. *P < 0.05

To investigate whether the effect of melatonin on chondrocyte hypertrophy is mediated by membrane receptors, we examined the expressions of the melatonin membrane receptors MT1 and MT2 in C3H10T1/2 cells. Representative immunofluorescence images are presented in Fig. 4a. Furthermore, the mRNA levels of hypertrophic genes including COL10A1, RUNX2, IHH, and PTHrP-R were reduced after treatment with luzindol (a blocker of the melatonin receptor) in melatonin-mediated hypertrophic differentiation (Fig. 4b–e). These data demonstrated that melatonin-mediated hypertrophic differentiation is mediated via melatonin membrane receptors.

### Melatonin activates Wnt-target genes during hypertrophic differentiation of BMSC-derived chondrocytes

**Fig. 5:**
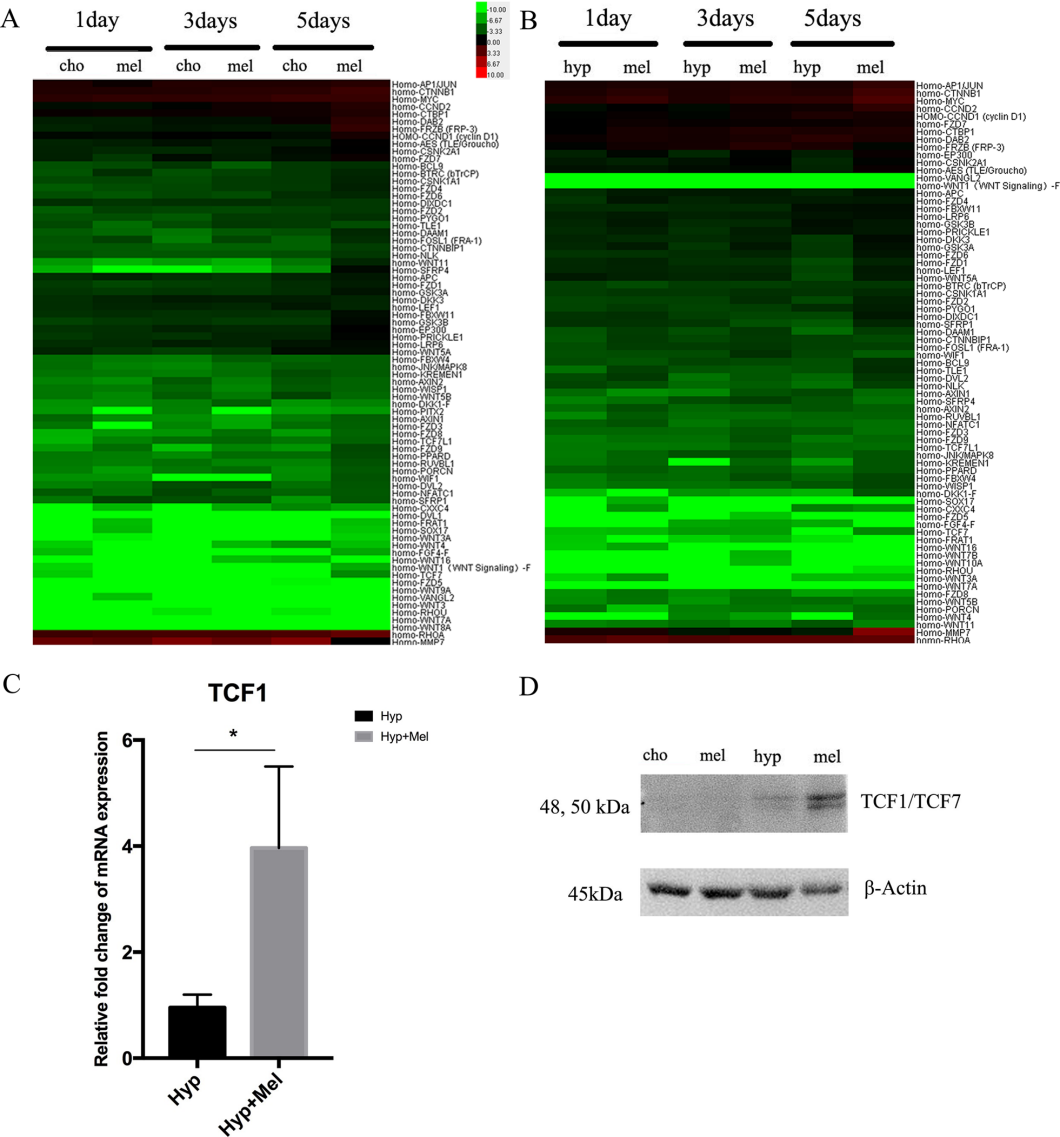
Melatonin activates Wnt-target genes during hypertrophic differentiation of BMSC-derived chondrocytes. **a** qRT-PCR array results in MSC-derived chondrocytes with or without 100 nM melatonin treatment for 1, 3, and 5 days without hypertrophic inducement. **b** The expression of downstream genes of the Wnt/β-catenin signaling pathway in MSC-derived chondrocytes during hypertrophic differentiation with or without 100 nM melatonin treatment for 1 day, 3 days, and 5 days. **c** The mRNA expression of TCF1 in the MSC-derived chondrocytes during hypertrophic differentiation. Gene expression levels were normalized to GAPDH mRNA. **d** The protein expression of TCF1/TCF7 in MSC-derived chondrocytes. *P < 0.05

To elucidate the molecular mechanism underlying melatonin-mediated hypertrophic differentiation, we conducted a qRT-PCR array analysis after melatonin treatment for 1, 3, and 5 days with or without hypertrophic inducement. We found that the expressions of multiple target genes of the Wnt/β-catenin signaling pathway were elevated after melatonin treatment (Fig. 5a, b), especially in the melatonin treatment group at day 5. TCF/LEF, a crucial transcription factor of the Wnt/β-catenin signaling pathway, was up-regulated at both the mRNA level and protein level during hypertrophic differentiation in the presence of melatonin, indicating that melatonin-mediated hypertrophic differentiation might be facilitated through Wnt/β-catenin-related genes (Fig. 5c, d).

### Melatonin promotes the nuclear translocation of β-catenin in C3H10T1/2 cells

**Fig. 6:**
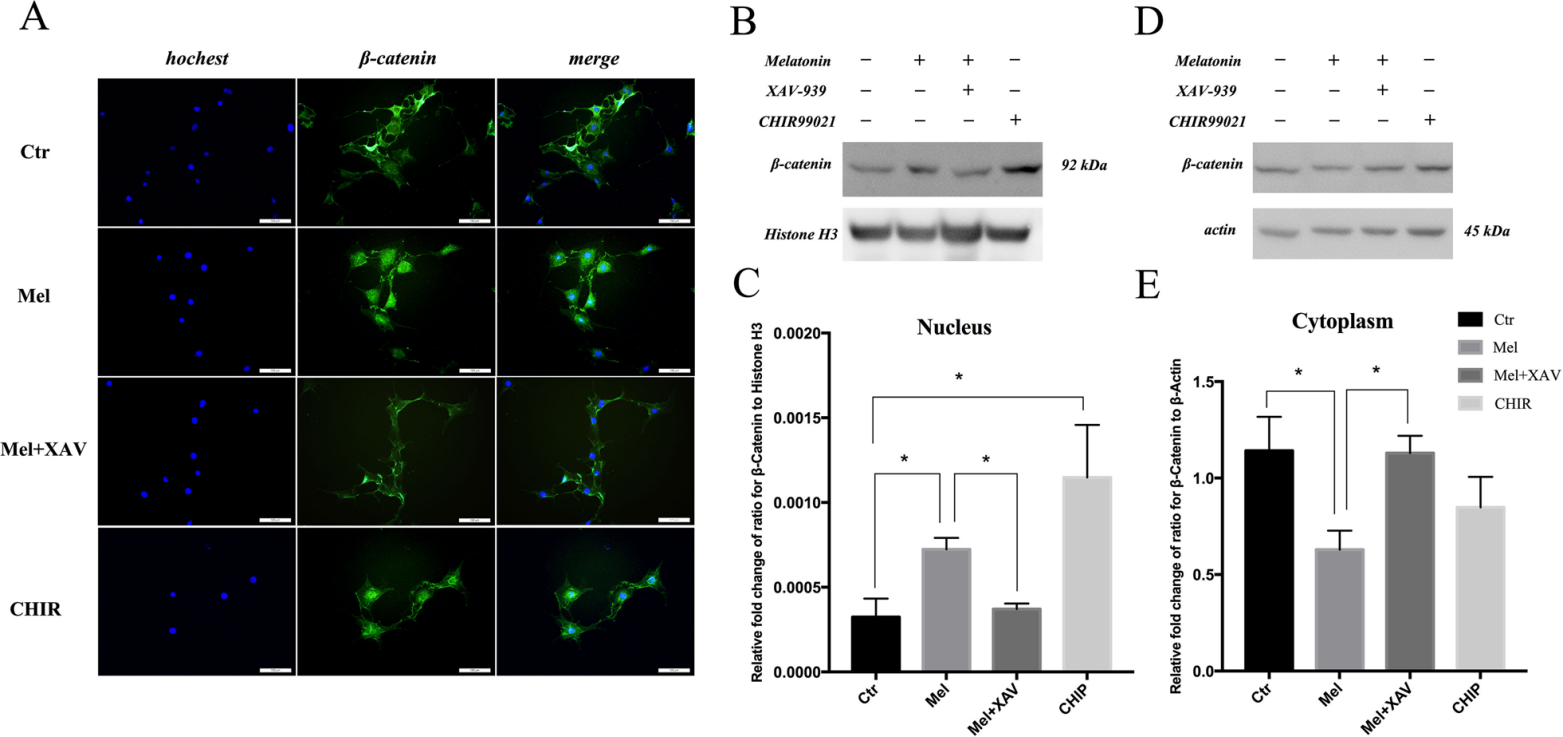
Melatonin promotes the nuclear translocation of β-catenin in the C3H10T1/2 cell line. **a** Immunofluorescence staining of β-catenin in C3H10T1/2 cells treated with or without 100 nM melatonin, the Wnt/β-catenin signaling pathway inhibitor XAV-939 or the agonist CHIR-90021. Scale bar = 100 μm. **b**, **d** Expression of β-catenin in nuclear and cytoplasmic components of cells treated as indicated. **c**, **e** Quantitative analyses of β-catenin expression levels in nuclear and cytoplasmic components from the cells from **b** and **d**. *P < 0.05

β-catenin is a crucial protein that translocates from the cytoplasm into the nucleus when Wnt signaling is activated. Immunofluorescence staining of β-catenin in C3H10T1/2 cells stimulated by melatonin is shown in Fig. 6a. Nuclear accumulation of fluorescence was observed in the melatonin group and the group treated with CHIR-90021, an agonist of the Wnt pathway. Western blot revealed that the nuclear β-catenin level was up-regulated, while the cytoplasmic β-catenin level was down-regulated after melatonin stimulation, and these changes were reversed by XAV-939, an inhibitor of the Wnt/β-catenin signaling pathway (Fig. 6b–e).

### Melatonin-mediated hypertrophic differentiation through Wnt/β-catenin signaling

**Fig. 7:**
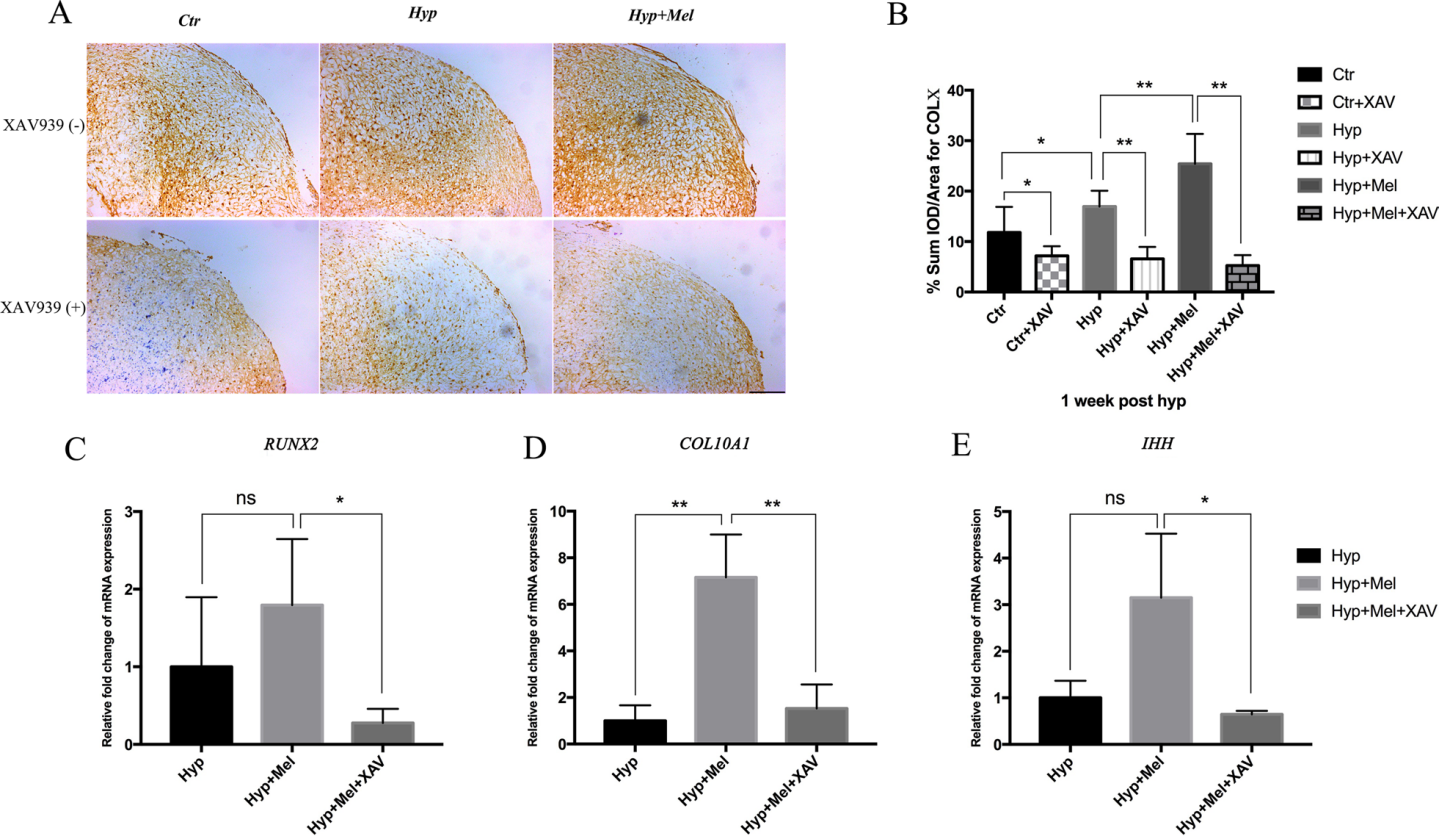
Wnt/β-catenin signaling pathway inhibitor blocks melatonin-mediated enhancement of hypertrophy. **a** Immunohistochemical staining of Col-X in MSC-derived cartilage. Scale bar = 200 μm. **b** Quantitative analysis of Col-X staining of the MSC-derived cartilage. **c**–**e** The mRNA expressions of RUNX2 (**c**), COL10A1 (**d**), and IHH (**e**) in the C3H10T1/2-derived chondrocytes. Gene expressions were normalized to that of GAPDH mRNA. *P < 0.05, **P < 0.01

Our data revealed that melatonin promoted nuclear translocation of β-catenin and activated Wnt/β-catenin pathway target gene expression. We next examined whether these events are related to the hypertrophic differentiation of chondrocytes. We next examined hypertrophic characteristics of the chondrocytes treated with melatonin after modulation of Wnt/β-catenin signaling using XAV-939. The results showed that the melatonin-enhanced expression of COL-X was partially reversed by XAV-939 (Fig. 7a). IOD analysis is shown in Fig. 7b. Gene expressions of RUNX2, COL10A1, and IHH were also decreased in melatonin-induced hypertrophic chondrocytes treated with XAV-939 (Fig. 7c–e).

## Discussion

Our previous studies of melatonin orchestrating BMSC chondrogenesis showed that hypertrophy-related genes in the melatonin treatment group were significantly increased compared with those in the control group at a later differentiation stage [22]. In this study, we identified melatonin as a promoter of hypertrophy differentiation of MSC-derived chondrocytes. Our data revealed that melatonin promotes MSC-derived chondrocyte hypertrophic differentiation via melatonin membrane receptors, followed by the activation of the Wnt/β-catenin signaling pathway.

Intramembranous ossification and endochondral ossification are two essential processes for bone formation and contribute to skeletal architecture and bone building [27]. Intramembranous ossification is responsible for the rudimentary development of cranial bones and also plays a role in bone fracture healing, in which MSCs directly develop into osteoblasts without cartilage participation. Unlike during intramembranous bone formation, in EO, cartilage acts as a transitional form. EO is responsible for the skeletal development of the trunk and limbs in vertebrates.

In previous studies, melatonin was found to be involved in the cell fate choice of BMSC differentiation into osteoblasts rather than adipocytes [28–30]. These findings revealed the anti-osteoporosis action of melatonin. Melatonin is also regarded as a bio-activator for bone repair because of its anti-oxygenation ability in the early stage of bone repair and its angiogenesis-promoting ability in the late stage of bone repair [31, 32].

Studies have shown that melatonin promoted the production of the cartilage matrix in porcine articular chondrocytes and up-regulated the chondrocyte hallmark Col-II rather than the hypertrophic biomarker Col-X [23]. Our previous study showed that melatonin enhanced the chondrogenesis of human BMSCs, partially via melatonin membrane receptors [22]. We found that melatonin up-regulated the expression of the hypertrophy-related genes COL10A1 and RUNX2 in human BMSC-derived chondrocytes in chondrogenic medium [22]. Melatonin is considered to have a protective effect on cartilage homeostasis because of its resistance against inflammation and oxidization in the maintenance of cartilaginous joints. Guo et al. reported that melatonin reduces matrix metalloproteinase production via the inhibition of Sirtuin-1-dependent NAMPT and NFAT5 signaling in chondrocytes [33]. Melatonin combined with treadmill exercise may have both preventive and synergistic effects on cartilage degeneration and is more effective in its initial phase.

Hypertrophic chondrocytes are recognized as the greatest contributors to the bone growth rate because of the expansion of cell volume in the growth plate. These cells are also excellent predictors of joint degenerative diseases, such as osteoarthritis, which is an age-related disorder that causes the loss of cartilage in joints. Although hypertrophic chondrocytes in the growth plate share high similarities with those in degenerative joints in terms of gross morphology, these cells are not exactly the same. Cooper et al. reported that chondrocytes in the growth plate undergo three phases for hypertrophic differentiation: Phase 1, true hypertrophy, is characterized by dry mass production that proportionately increases with fluid uptake; phase 2, cell swelling, is characterized by fluid uptake that increases proportionately and greater than dry mass production; and phase 3, another true hypertrophy, is controlled by the IGF1 signaling pathway [7]. In contrast to growth plate chondrocytes, articular chondrocytes increase in volume by extracellular matrix degradation and collagen network damage, leading to hypo-osmotic swelling in the initial stage of osteoarthritis [34–37].

A previous study reported that melatonin treatment reduced the mRNA levels of hypertrophic markers such as COL10A1 and ALP in the growth plate chondrocytes [38]. Our present data, acquired from both human BMSC and C3H10T1/2 cell analysis, demonstrated that melatonin up-regulated the hallmarks of hypertrophic chondrocytes, such as COL10A1, RUNX2, and ALP. Two opposite functions of melatonin were observed, which may be from the cellular heterogeneity of different chondrocytes. Growth plate chondrocytes are composed of rest, proliferative, and hypertrophic chondrocytes, which are differentiated cells with a high level of hypertrophy markers. Therefore, melatonin treatment cannot further enhance the expression of the hypertrophic hallmarks for these mature chondrocytes. Chondrocytes from MSCs, which are cells with multi-lineage differentiation potentials, showed favorable ability for hypertrophic differentiation in a micromass culture system [39], and therefore, our results provided completely different information in effect of melatonin on the chondrocytes differentiation. The IHH/PTHrP negative feedback loop acts as a pivotal effector of EO. IHH, expressed in pre-hypertrophic chondrocytes, promotes the production of PTHrP in proliferative chondrocytes. PTHrP suppresses IHH expression by binding with PTHrP receptors, which are expressed on the membrane of hypertrophic chondrocytes, leading to an outcome that favors chondrocyte proliferation over hypertrophic differentiation. Our results showed that melatonin enhanced IHH and PTHrP-R expression and suppressed PTHrP in human BMSC-derived chondrocytes. Collectively, our current results indicate that melatonin acts as a vital promoter of EO by regulating chondrocyte hypertrophy during MSC differentiation.

The hypertrophic differentiation of chondrocytes is regulated by many cellular pathways, and previous studies have shown that the classical Wnt pathway is involved in this process. The Wnt pathway is divided into classical and non-classical Wnt pathways according to whether the downstream pathways require β-catenin parameters [40–42]. Both classical and non-classical Wnt pathways play an important role in the development of bone and cartilage. Many Wnt proteins have been found in the cartilage growth plate [43]. Hartmann et al. found that Wnt5b/11 was expressed in pre-hypertrophic chondrocytes in chicken limb buds, while Wnt4 was not expressed in hypertrophic chondrocytes [44]. Rudnicki et al. also found that the abnormal distribution of Wnt7 led to limb chondrodysplasia in chickens [45]. Similar studies have shown that abnormalities in Wnt5a/5b hinder limb cartilage hypertrophy and differentiation [43, 44]. Notably, Wnt proteins are not expressed in resting and proliferative chondrocytes but are mainly expressed in hypertrophic and pre-hypertrophic chondrocytes [44]. These Wnt proteins primarily originate from the perichondrium and osteoblasts in the surrounding cancellous bone. In this study, after chondrocytes derived from stem cells were cultured in 3D and hypertrophy was induced by the addition of thyroxine T3, gene chip analysis showed no significant difference in the expression of Wnt5a/5b and Wnt4 proteins in these cells; however, a decrease in the gene expression of Wnt11 and no detecting of other Wnt genes were observed. This may be because Wnt proteins mainly originate from the perichondrium and osteoblasts around the growth plate in vivo but do not originate from chondrocytes.

XAV-939 is an inhibitor of the terminal anchor polymerase (tankyrase) that accelerates the degradation of β-catenin by stabilizing the axis inhibitor Axin, thus affecting the nuclear translocation of β-catenin and regulating the transcription of target genes downstream of the ganglion [46]. In this study, the hypertrophic effect of melatonin on cartilage was inhibited by XAV-939. Immunohistochemistry revealed that expression of Col-X was lower and the extent of staining was smaller in the melatonin + XAV939 group compared with the melatonin group. This suggests that melatonin promotes the hypertrophy of MSC-derived chondrocytes by regulating the nuclear translocation of β-catenin. Western blot showed that the nuclear expression of β-catenin increased significantly and cytoplasmic β-catenin decreased after the addition of melatonin, and these events were reversed by treatment with XAV939. These findings suggested that melatonin promotes the nuclear translocation of β-catenin.

In summary, our results indicate that the molecular mechanism by which melatonin promotes hypertrophy and differentiation of MSC-derived chondrocytes involves activation of the Wnt/β-catenin signal pathway and increasing the nuclear translocation of β-catenin.

This study had several limitations. First, we used MSCs isolated from human bone marrow, which fulfills the criteria proposed by the ISCT [26]. However, MSCs isolated according to ISCT criteria can produce heterogeneous, non-clonal cultures of stromal cells containing stem cells with different multipotential properties, committed progenitors, and differentiated cells [47, 48]. Therefore, further studies are needed to explore whether melatonin contributes to the hypertrophic differentiation of chondrocytes derived from special subpopulations of MSCs or MSCs isolated from other tissues, such as adipose tissue, umbilical cord, and dental pulp. Second, our results demonstrating the effects of melatonin on chondrocyte terminal differentiation were derived from in vitro studies with human MSCs and the C3H10T1/2 cell line. In vivo studies are required to confirm these preliminary findings. Finally, this study demonstrates the link between melatonin and Wnt/β-catenin in hypertrophic differentiation of chondrocytes. The connection between melatonin and Wnt/β-catenin was previously shown in osteogenesis [49] and cancer metastasis [50]. Although these studies suggest a functional relationship between melatonin and Wnt/β-catenin that may be involved in various biological processes, the precise mechanism how melatonin activates the Wnt/β-catenin pathway had not been explored. Therefore, further studies revealing the specific mechanism by which Wnt/β-catenin is activated by melatonin are needed.

## Conclusions

Our results suggest that melatonin enhances the hypertrophic differentiation of MSC-derived chondrocytes through the Wnt signaling pathway. These findings provide evidence establishing the role of melatonin in the regulation of MSC differentiation and bone development. Our results may facilitate the clinical application of melatonin in the treatment of skeletal developmental diseases.

## Supplementary Information

**Additional file 1.** Expression of surface markers in BMSCs. The third passage of primary isolated BMSCs was subjected to flow cytometry to investigate the expression of CD105, CD73, CD34, CD 45, and HLA. These results were representative of three independent experiments.

**Additional file 2.** The effect of melatonin on the viability and proliferation of BMSCs. The third passage of primary isolated BMSCs was cultured with or without melatonin treatment at different concentrations for 24, 48 h and then were subjected to CCK8 assay. The absorbance at 450 nm was measured at each time point. The results were representative of three independent experiments, and the data were presented as mean ± SD. Ns, no significance versus control group at different time points.

**Additional file 3.** Flow cytometry analysis of apoptosis with Annexin V/PI staining. The third passage of BMSCs was treated with different concentrations of melatonin for 48 h and then were collected to perform flow cytometry Apoptosis Assay. The percentage of apoptotic cells was showed in the histogram, presenting the early (FITC+/PI−), late (FITC+/PI+), and total apoptotic cells, respectively. These results (**a**–**d**) were representative pictures of three independent experiments. *P < 0.05, **P < 0.01, ns was no significance versus the corresponding control group at each apoptotic stage.

**Additional file 4.** Cell senescence assay with β-galactosidase staining. **a** The third passage of BMSCs was treated with different concentrations of melatonin for 48 h and then were collected to β-galactosidase staining. The senescent cells were dyed blue in each treatment group, scale bar = 100 μm. **b** The percentage of senescent cells was calculated with Image J. *P < 0.05 versus control, **P < 0.01 versus control.

**Additional file 5.** The effect of CHIR99021 on the hypertrophic differentiation of BMSCs-derived chondrocytes. The third passage BMSCs were under chondrogenic inducement for two weeks, and then hypertrophic inducement (with or without 5 μM CHIR99021) for one week, the cells were collected and collected to perform RT-PCR analysis to investigate the expression of collagen type X (COL10A1) (**a**), alkaline phosphatase (ALP) (**b**), runt-related transcription factor 2 (RUNX2) (**c**), and Indian hedgehog (IHH) (**d**). Relative gene expression levels were calculated by using the 2^−△△Ct^ method. *P < 0.05, **P < 0.01, ns was no significance versus the corresponding control group.

**Additional file 6.** Supplementary methods and materials.

## Data Availability

The data sets used and analyzed during the current study are available from the corresponding author upon reasonable request.

## Abbreviation

MSCs: Mesenchymal stem cells
COL10A1: Type X collagen
ALP: Alkaline phosphatase
RUNX2: Runt-related transcription factor 2
IHH: Indian hedgehog
PTHrP-R: Parathyroid hormone-related protein receptor
PTHrP: Parathyroid hormone-related protein
EO: Endochondral ossification
TCF: T cell-specific factor
LEF: Lymphoid enhancer binding protein
BMSC: Bone marrow mesenchymal stem cell
DMEM: Dulbecco’s modified Eagle’s medium
FBS: Fetal bovine serum
PCR: Polymerase chain reaction
TGF-β3: Transforming growth factor–β3
COLX: Collagen type X
GAPDH: Glyceraldehyde-3-phosphate dehydrogenase
Col-II: Type II collagen
Col-X: Type X collagen
IOD: Integral optical density
qRT-PCR: Quantitative real-time polymerase chain reaction
TCF/LEF: T-cell factor/lymphoid enhancer factor
GPC: Growth plate chondrocyte
HBMSC: Human bone marrow mesenchymal stem cell
MMP13: Matrix metalloproteinase 13
