# Supplementary figures and images for: Melatonin contributes to the hypertrophic differentiation of mesenchymal stem cell-derived chondrocytes via activation of the Wnt/β-catenin signaling pathway: Melatonin promotes MSC-derived chondrocytes hypertrophy
Source: Stem Cell Res Ther. 2021 Aug 21;12:467. doi: 10.1186/s13287-021-02536-x (PMC8379782; doi:10.1186/s13287-021-02536-x)

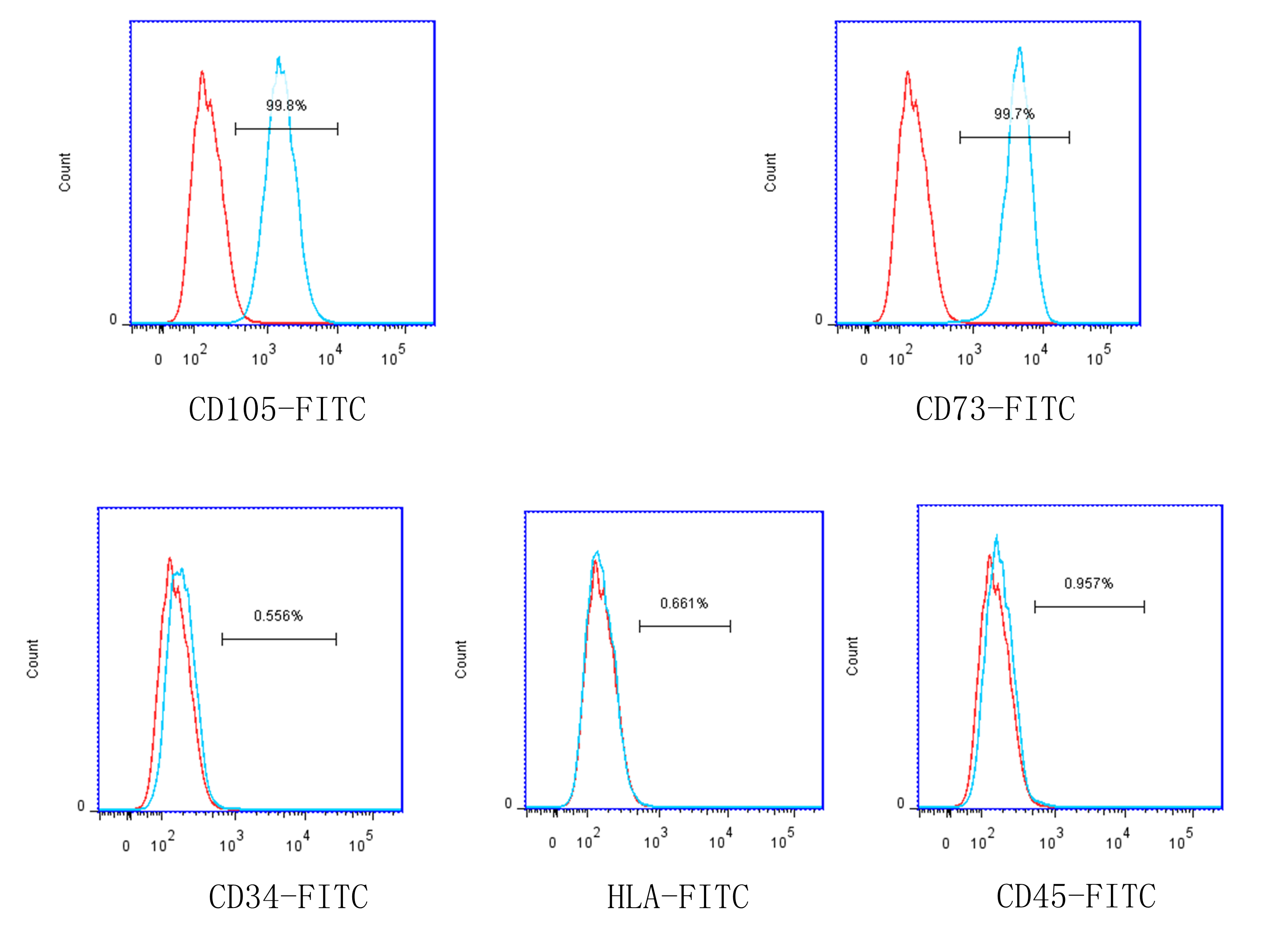

Supplement: Supplementary file 1 — Additional file 1. Expression of surface markers in BMSCs. The third passage of primary isolated BMSCs was subjected to flow cytometry to investigate the expression of CD105, CD73, CD34, CD 45, and HLA. These results were representative of three independent experiments. [file 13287_2021_2536_MOESM1_ESM.tif]

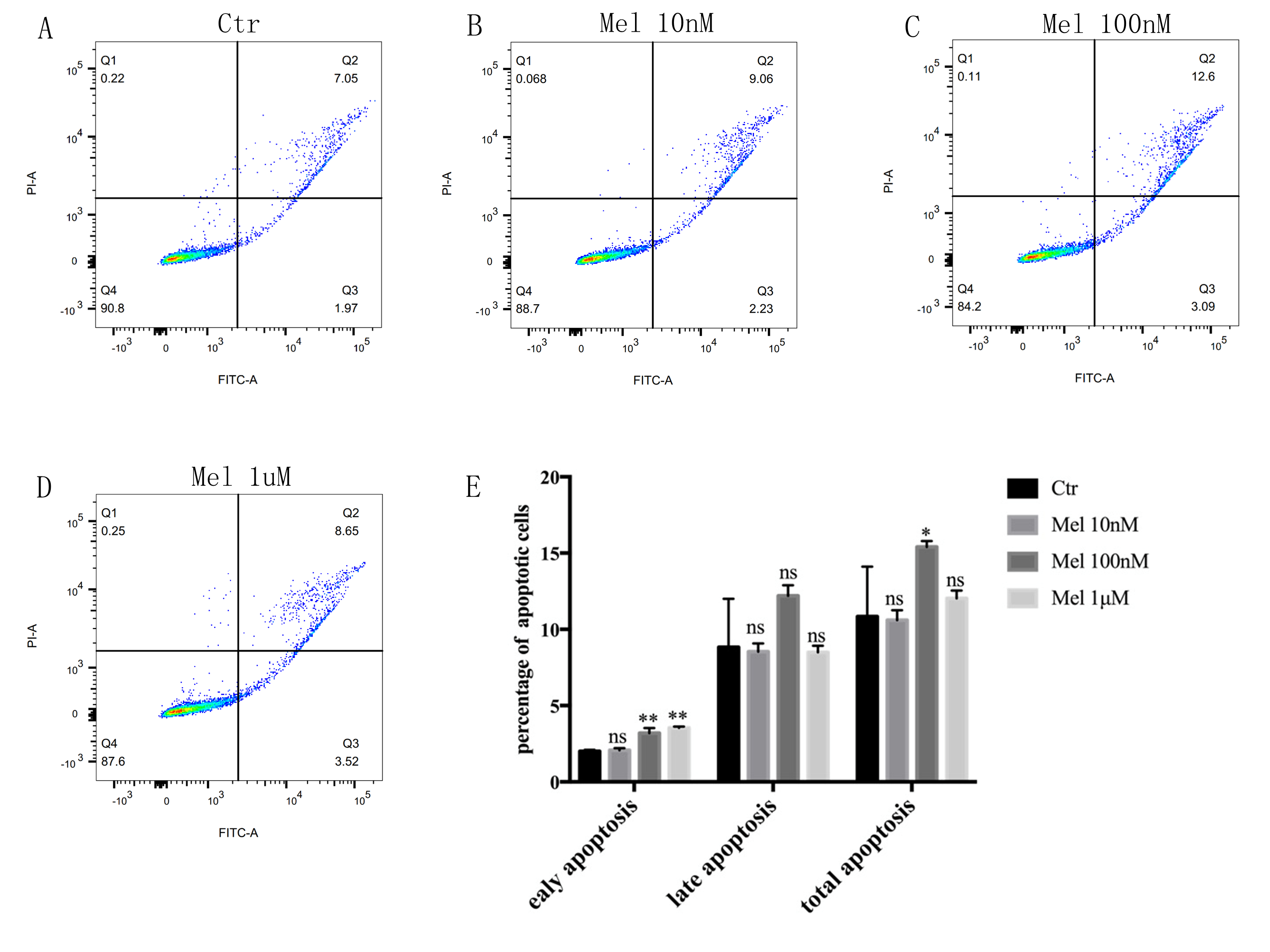

Supplement: Supplementary file 3 — Additional file 3. Flow cytometry analysis of apoptosis with Annexin V/PI staining. The third passage of BMSCs was treated with different concentrations of melatonin for 48 h and then were collected to perform flow cytometry Apoptosis Assay. The percentage of apoptotic cells was showed in the histogram, presenting the early (FITC+/PI−), late (FITC+/PI+), and total apoptotic cells, respectively. These results (a–d) were representative pictures of three independent experiments. *P < 0.05, **P < 0.01, ns was no significance versus the corresponding control group at each apoptotic stage. [file 13287_2021_2536_MOESM3_ESM.tif]

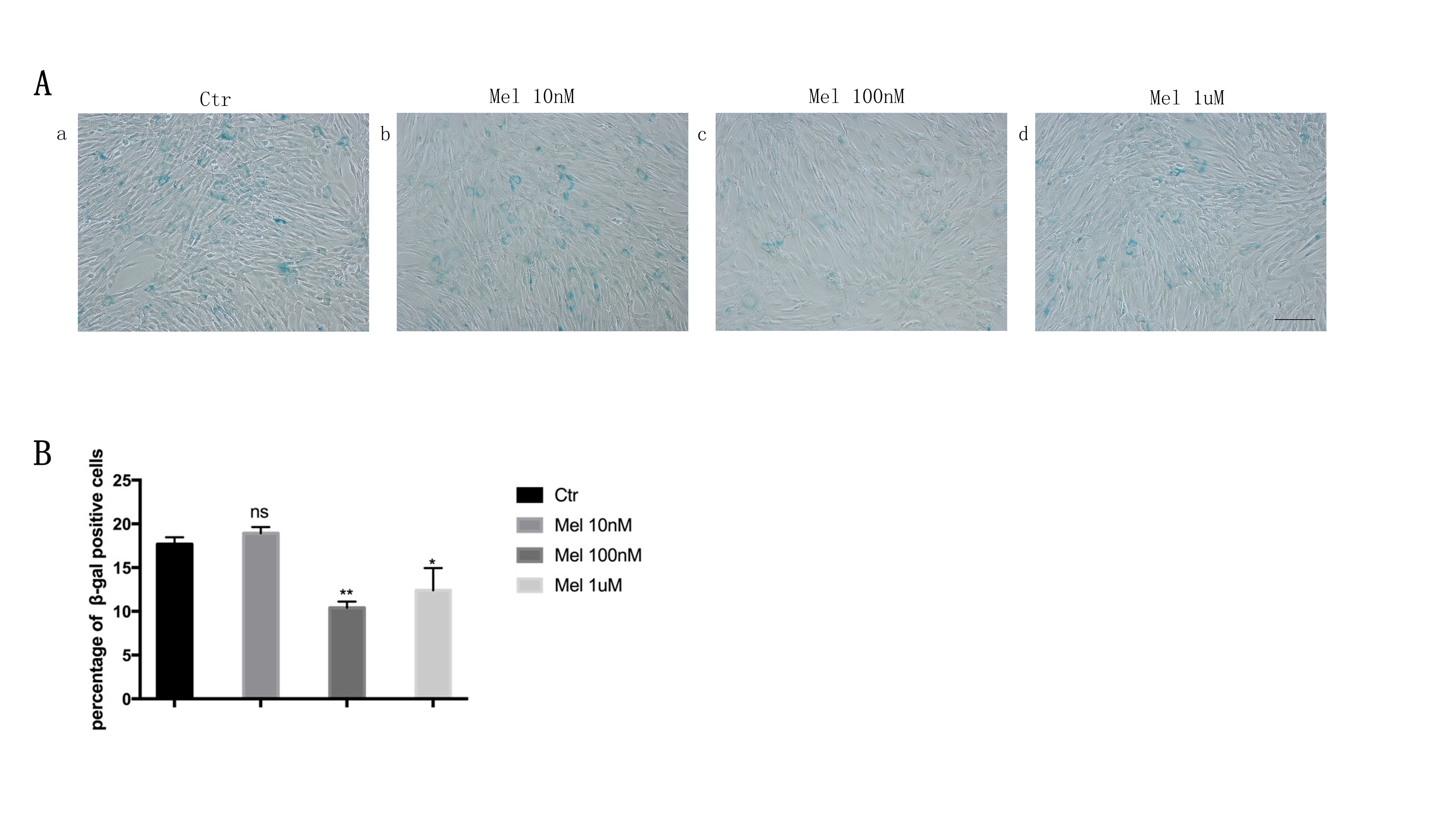

Supplement: Supplementary file 4 — Additional file 4. Cell senescence assay with β-galactosidase staining. a The third passage of BMSCs was treated with different concentrations of melatonin for 48 h and then were collected to β-galactosidase staining. The senescent cells were dyed blue in each treatment group, scale bar = 100 μm. b The percentage of senescent cells was calculated with Image J. *P < 0.05 versus control, **P < 0.01 versus control. [file 13287_2021_2536_MOESM4_ESM.tif]
