# Supplementary material for: Melatonin contributes to the hypertrophic differentiation of mesenchymal stem cell-derived chondrocytes via activation of the Wnt/β-catenin signaling pathway: Melatonin promotes MSC-derived chondrocytes hypertrophy
Source: Stem Cell Res Ther. 2021 Aug 21;12:467. doi: 10.1186/s13287-021-02536-x (PMC8379782; doi:10.1186/s13287-021-02536-x)
Supplement: Supplementary file 6 — Additional file 6. Supplementary methods and materials. [file 13287_2021_2536_MOESM6_ESM.docx]

**Additional file 6: Supplementary methods and materials**

**Phenotype identification of BMSCs**

The cells were isolated and cultured from the human bone marrow following the method in the manuscript. The third passage cells were used for this experiment. The cells were digested with trypsin at 80% conﬂuence. Then the digestion was terminated by 10%FBS LD-DMEM complete medium. The cells were washed there times by PBS. Cells were collected after centrifugation, and each 3×10^5^ cells were reacted with anti- CD105, CD73, CD34, HLA, and CD45 antibodies (Abcam, USA) at room temperature for 30 min respectively. Then, they were washed twice with PBS and reacted with FITC-labeled secondary antibody (Abcam, USA) for 15 minutes. After being resuspended with PBS, the washed cells were detected and analyzed by flow cytometry (BD FACSCalibur, USA).

**Cell Proliferation and Cytotoxicity Assay by CCK8**

The third passage BMSCs were cultured following the above method with or without melatonin at different concentrations (0, 10nM, 100nM, 1μM) for 24 or 48 hours. Inoculate cell suspension (100 μl/well) in a 96-well plate. Pre-incubate the plate in a humidified incubator (at 37°C, 5% CO2). Add 10 μl of the CCK-8 solution (Beyotime, China) to each well of the plate. Incubate the plate for 4 hours in the incubator and then measure the absorbance at 450 nm using a microplate reader (Thermo, USA).

**Apoptosis Assay by flow cytometry: Annexin V/PI double staining**

The third passage BMSCs were cultured following the above method with or without melatonin at different concentrations (0, 10nM, 100nM, 1μM) for 48 hours, then were digested and suspended. The suspended cells were directly collected in a 10ml centrifuge tube, and the number of cells in each sample was (1-5) × 10^6^. Centrifuge for 5min from 500 to 1000r/min, and the culture medium was discarded. Then wash with incubation buffer and centrifuge at 500~1000r/min for 5min. The cells were resuspended with 100ul of labeled solution and incubated at room temperature for 10-15 min in the dark. Centrifuged at 500-1000r /min for 5min to precipitate the cells and washed with incubation buffer for one time. Fluorescence (SA-flours) solution (Millipore, Italy) was added and incubated at 4℃ for 20min, avoiding light and vibration. The excitation wavelength of the flow cytometer was 488nm. FITC fluorescence was detected by a passband filter with a wavelength of 515nm, and propidium iodide (PI) was detected by another filter with a wavelength greater than 560nm.

**In situ senescence-associated β-galactosidase assay**

The third passage BMSCs were cultured following the above method with or without melatonin at different concentrations (0, 10nM, 100nM, 1μM) for 48 hours. The X-gal staining was detected using the in situ β-galactosidase staining kit (Beyotime, China). According to the manufacturer's instructions, the adherent cells were cultured in a 6-well plate for 48 hours, then washed with PBS three times after the cell culture medium was removed. 1ml of β-galactoside staining fixation solution was added and fixed at room temperature for 15 minutes. The cell fixation solution was removed, the cells were washed with PBS three times afterward, with 3 minutes each time. PBS was then sucked out, and 1 ml of the X-gal dye working solution was added to each well, which was incubated overnight at 37℃. The 6-well plate was covered by plastic wrap to prevent evaporation. Observation and counting were performed under an ordinary light microscope（Leica, Germany）the next day. The senescent cells were stained blue, β-galactosidase-positive cells percentage was calculated out of at least 500 cells in different microscope fields[1].

**RT-PCR analysis to verify the CHIR99021 treatment inducing hypertrophic differentiation of BMSCs-derived chondrocytes**

BMSCs chondrogenic differentiation and thereafter inducing hypertrophic differentiation with or without CHIR99021 treatment were described in the Materials and Methods of the manuscript. The expressions of the following genes were examined: type X collagen (COL10A1), alkaline phosphatase (ALP), Runt-related transcription factor 2 (RUNX2), Indian hedgehog (IHH). Relative expression levels for each primer set were expressed as fold changes by the 2^-△△Ct^ method.

**Results**

Flow cytometry was used for measuring the surface marker expression rates of the third passage cells from primary cells derived from the bone marrow in vitro. The expression rates of fluorescence were CD105 99.8%, CD73 99.7%, CD34 0.556%, HLA 0.661% and CD45 0.957%, respectively, which indicated that these cells were typical mesenchymal stem cells (**See Additional file 1**).

To determine the effect of melatonin on cells viability and proliferation, BMSCs were cultured with or without melatonin treatment at different concentrations for 24, 48 hours and then were subjected to CCK8 assay. The data showed that there was no significant difference between melatonin and control groups (**See Additional file 2**), indicating that melatonin treatment could not improve MSCs viability and proliferation at both nanomolar and micromolar levels.

To determine the effect of melatonin on cells apoptosis, BMSCs were cultured with or without melatonin treatment at different concentrations for 48 hours and then were subjected to a flow cytometric test. The data showed that different levels of melatonin had no significant effect on late apoptotic cells. Interestingly, the percentage of the early apoptotic cells significantly increased with 100nM and 1Μm melatonin treatment, compared with the control group. Besides, the 100nM melatonin treatment could significantly increase the ratio of the total cells apoptosis (**See Additional file 3**).

To verify the effect of melatonin on MSC senescence, the situ β-galactosidase assay was performed to reveal that the senescence process of BMSCs was noticeably inhibited after incubation with 100nM and 1μM melatonin comparing with the control group, while no significant difference was shown in the 10nM melatonin group (**See Additional file 4**).

To investigate whether CHIR90021 enhance hypertrophic differentiation of BMSC-derived chondrocytes, RT-PCR analysis was performed, and the results showed that the mRNA expression of hypertrophic genes such as COL10A1, ALP, RUNX2, IHH was up-regulated after 7-days treatment of CHIR99021 (**See Additional file 5**).

**Reference**

1. Alessio N, Stellavato A, Squillaro T, Del Gaudio S, Di Bernardo G, Peluso G, De Rosa M, Schiraldi C, Galderisi U: **Hybrid complexes of high and low molecular weight hyaluronan delay in vitro replicative senescence of mesenchymal stromal cells: a pilot study for future therapeutic application**. *Aging (Albany NY)* 2018, **10**(7):1575-1585.
